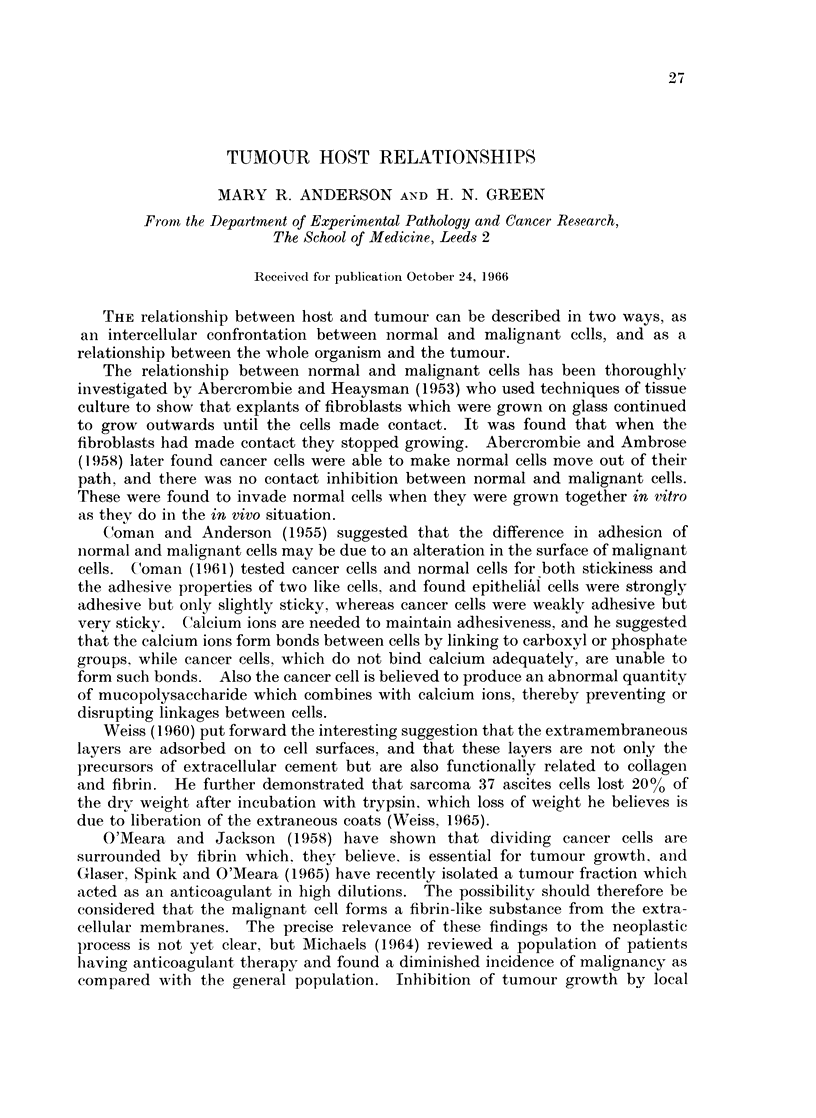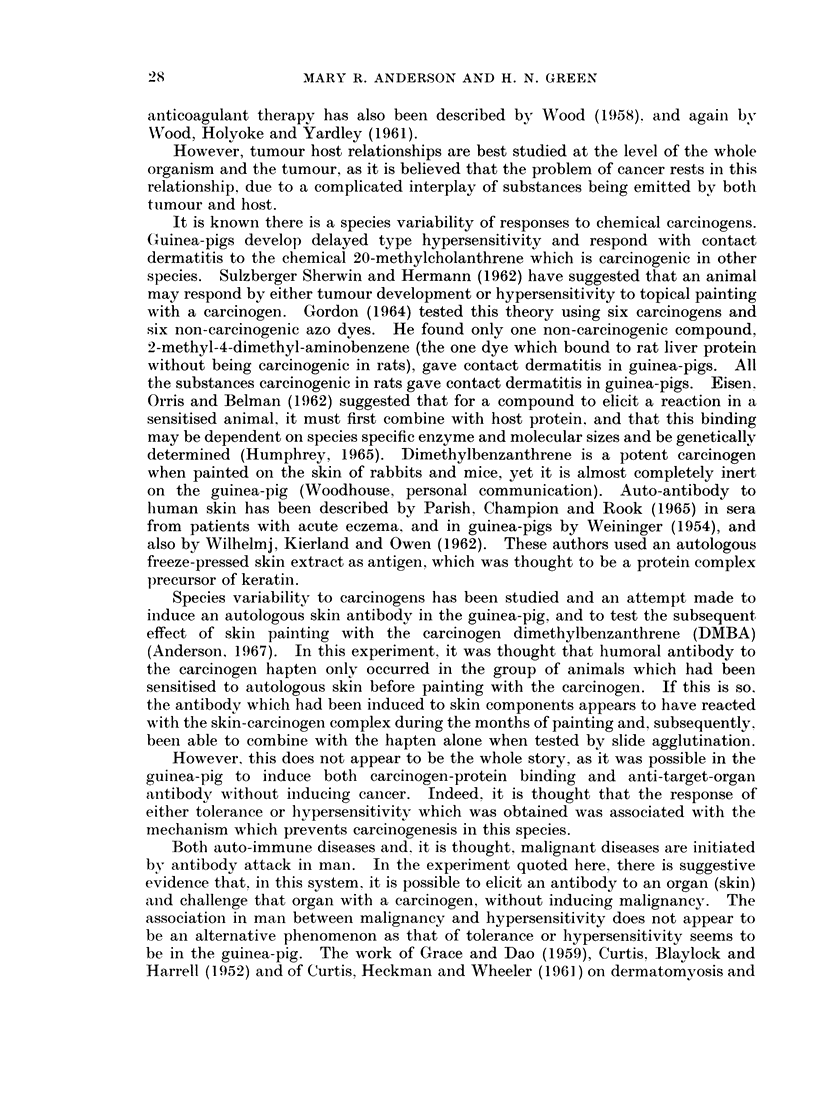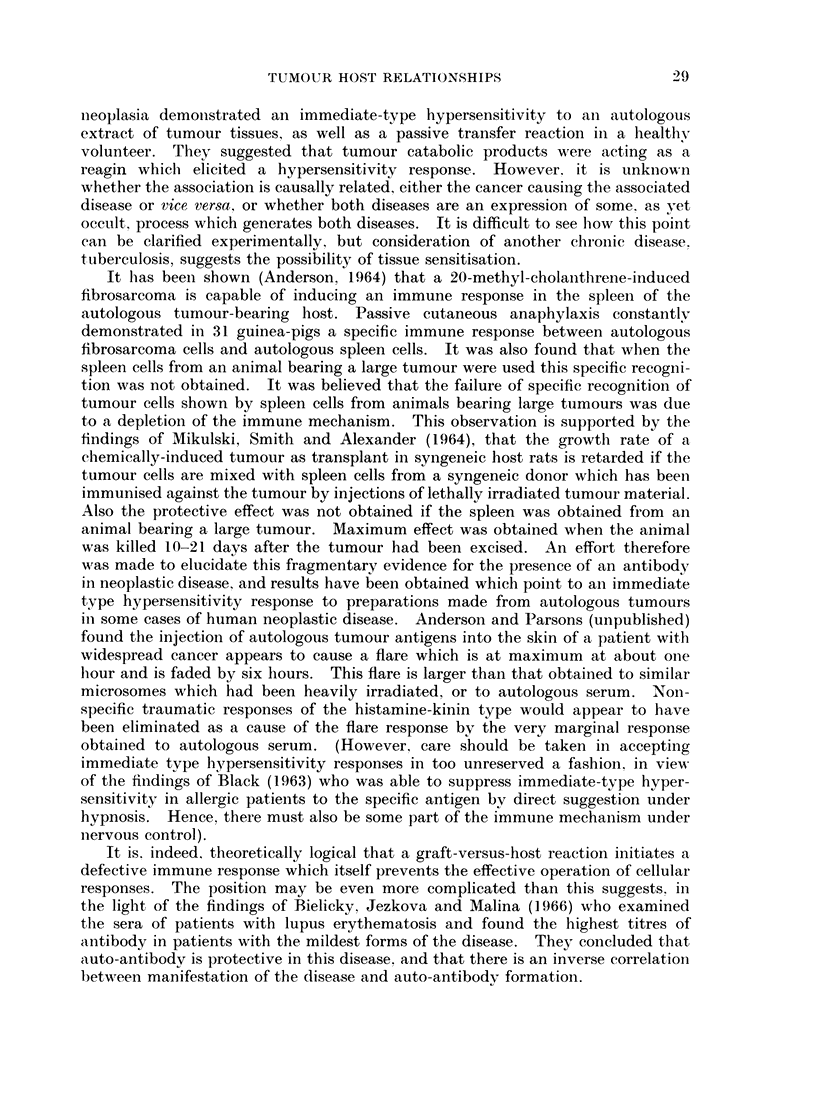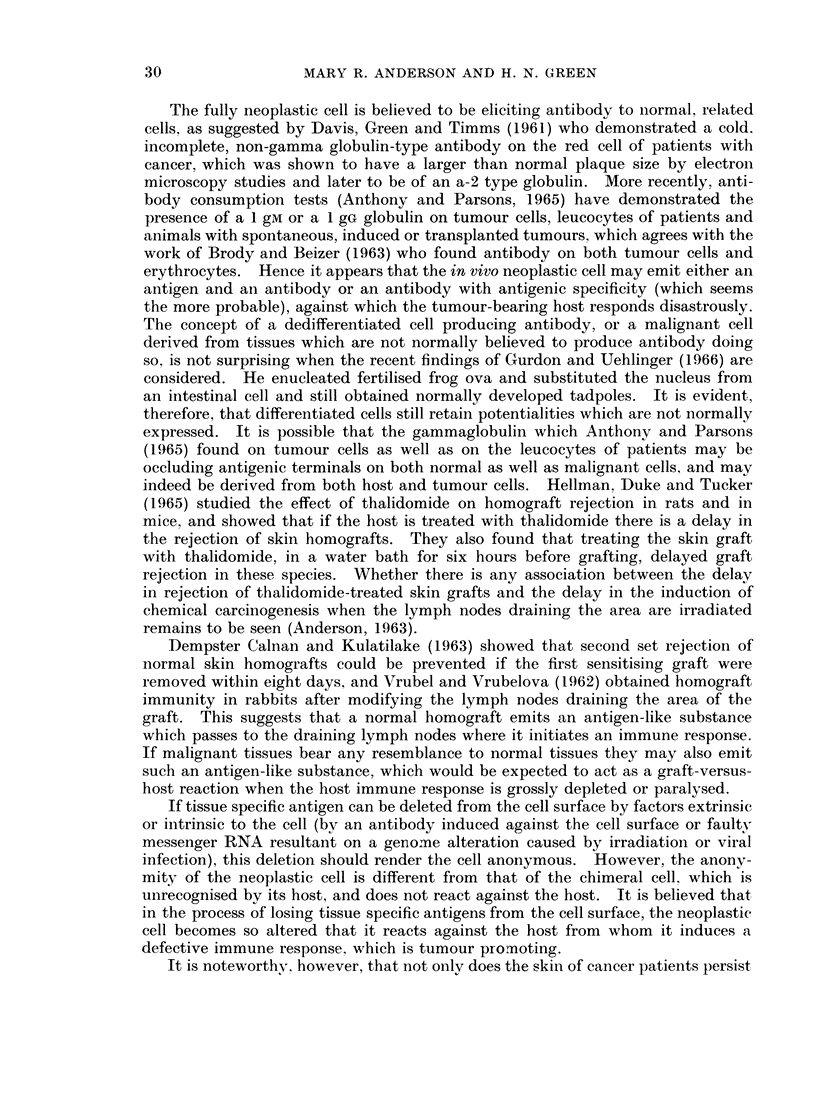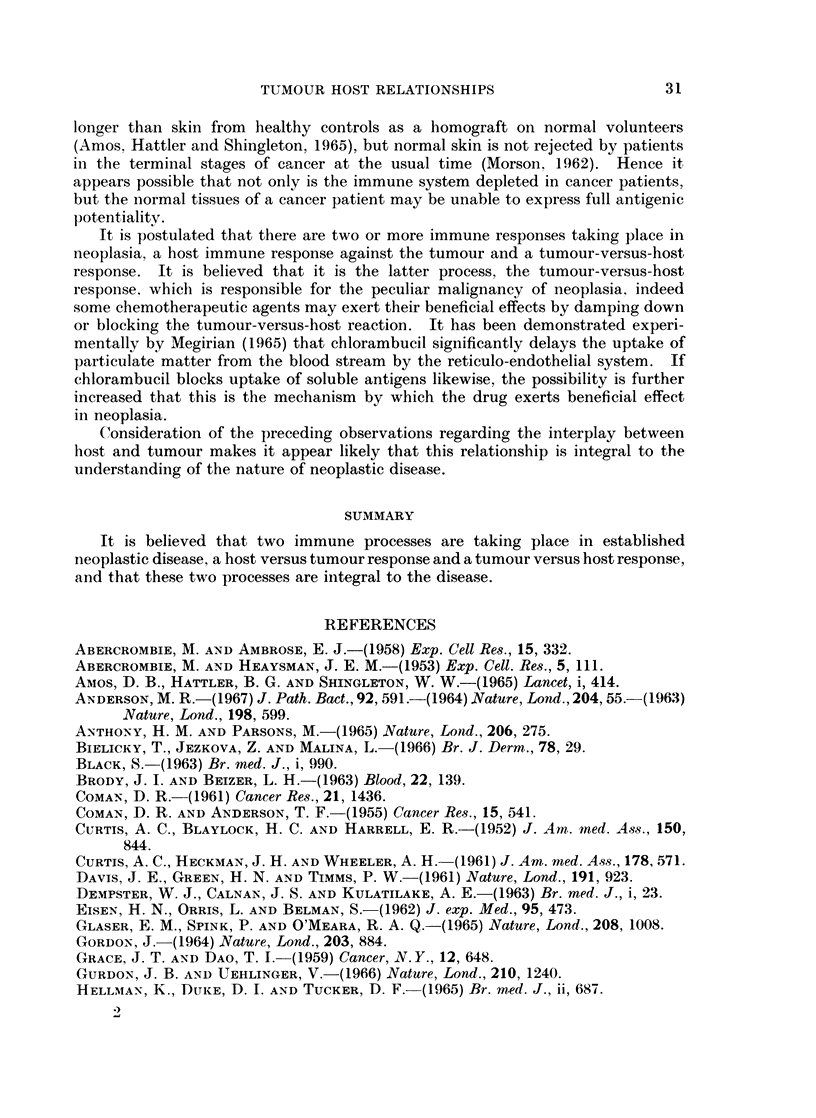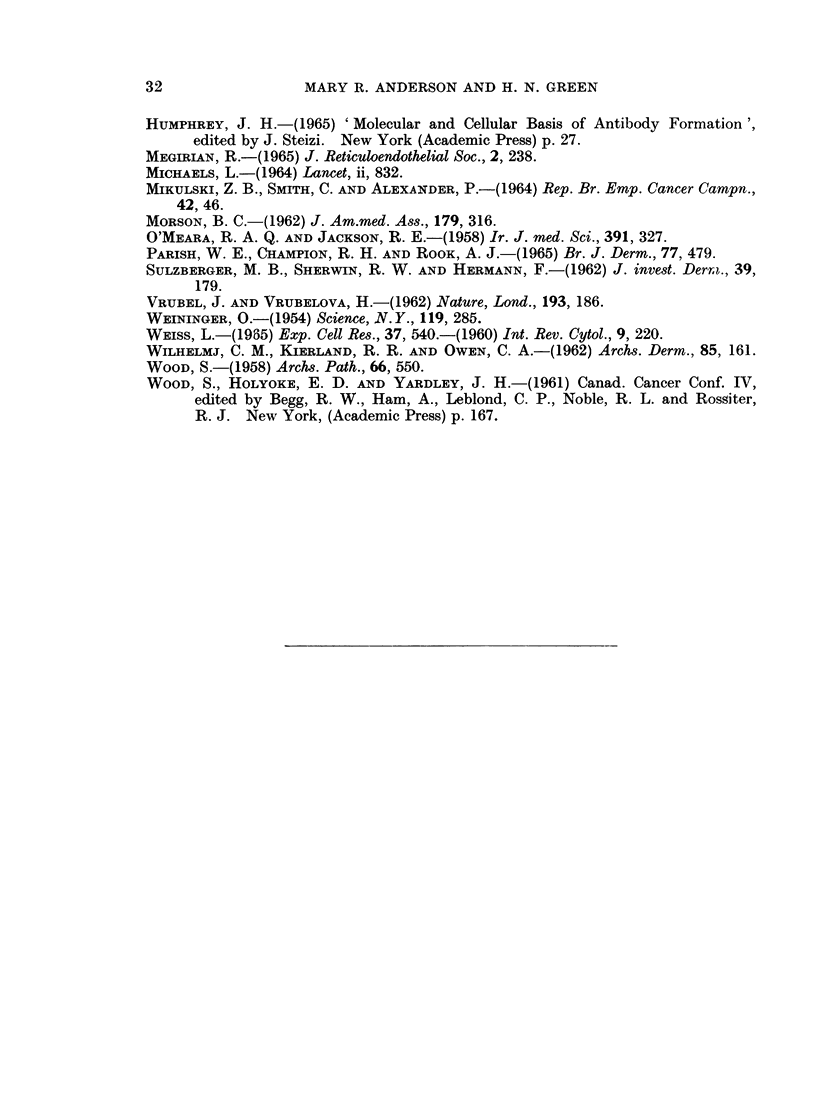# Tumour host relationships.

**DOI:** 10.1038/bjc.1967.4

**Published:** 1967-03

**Authors:** M. R. Anderson, H. N. Green


### TUMOUR HOST RELATIONSHIPS

MARY R. ANDERSON AND H. N. GREEN

*From the Department of Experimental Pathology and Cancer Research, The School of Medicine, Leeds 2*

Received for publication October 24, 1966

THE relationship between host and tumour can be described in two ways, as an intercellular confrontation between normal and malignant cells, and as a relationship between the whole organism and the tumour.

The relationship between normal and malignant cells has been thoroughly investigated by Abercrombie and Heaysman (1953) who used techniques of tissue culture to show that explants of fibroblasts which were grown on glass continued to grow outwards until the cells made contact. It was found that when the fibroblasts had made contact they stopped growing. Abercrombie and Ambrose (1958) later found cancer cells were able to make normal cells move out of their path, and there was no contact inhibition between normal and malignant cells. These were found to invade normal cells when they were grown together *in vitro* as they do in the *in vivo* situation.

Coman and Anderson (1955) suggested that the difference in adhesion of normal and malignant cells may be due to an alteration in the surface of malignant cells. Coman (1961) tested cancer cells and normal cells for both stickiness and the adhesive properties of two like cells, and found epithelial cells were strongly adhesive but only slightly sticky, whereas cancer cells were weakly adhesive but very sticky. Calcium ions are needed to maintain adhesiveness, and he suggested that the calcium ions form bonds between cells by linking to carboxyl or phosphate groups, while cancer cells, which do not bind calcium adequately, are unable to form such bonds. Also the cancer cell is believed to produce an abnormal quantity of mucopolysaccharide which combines with calcium ions, thereby preventing or disrupting linkages between cells.

Weiss (1960) put forward the interesting suggestion that the extramembraneous layers are adsorbed on to cell surfaces, and that these layers are not only the precursors of extracellular cement but are also functionally related to collagen and fibrin. He further demonstrated that sarcoma 37 ascites cells lost 20% of the dry weight after incubation with trypsin, which loss of weight he believes is due to liberation of the extraneous coats (Weiss, 1965).

O'Meara and Jackson (1958) have shown that dividing cancer cells are surrounded by fibrin which, they believe, is essential for tumour growth, and Glaser, Spink and O'Meara (1965) have recently isolated a tumour fraction which acted as an anticoagulant in high dilutions. The possibility should therefore be considered that the malignant cell forms a fibrin-like substance from the extracellular membranes. The precise relevance of these findings to the neoplastic process is not yet clear, but Michaels (1964) reviewed a population of patients having anticoagulant therapy and found a diminished incidence of malignancy as compared with the general population. Inhibition of tumour growth by local anticoagulant therapy has also been described by Wood (1958), and again by Wood, Holyoke and Yardley (1961).

However, tumour host relationships are best studied at the level of the whole organism and the tumour, as it is believed that the problem of cancer rests in this relationship, due to a complicated interplay of substances being emitted by both tumour and host.

It is known there is a species variability of responses to chemical carcinogens. Guinea-pigs develop delayed type hypersensitivity and respond with contact dermatitis to the chemical 20-methylcholanthrene which is carcinogenic in other species. Sulzberger Sherwin and Hermann (1962) have suggested that an animal may respond by either tumour development or hypersensitivity to topical painting with a carcinogen. Gordon (1964) tested this theory using six carcinogens and six non-carcinogenic azo dyes. He found only one non-carcinogenic compound, 2-methyl-4-dimethyl-aminobenzene (the one dye which bound to rat liver protein without being carcinogenic in rats), gave contact dermatitis in guinea-pigs. All the substances carcinogenic in rats gave contact dermatitis in guinea-pigs. Eisen, Orris and Belman (1962) suggested that for a compound to elicit a reaction in a sensitised animal, it must first combine with host protein, and that this binding may be dependent on species specific enzyme and molecular sizes and be genetically determined (Humphrey, 1965). Dimethylbenzanthrene is a potent carcinogen when painted on the skin of rabbits and mice, yet it is almost completely inert on the guinea-pig (Woodhouse, personal communication). Auto-antibody to human skin has been described by Parish, Champion and Rook (1965) in sera from patients with acute eczema, and in guinea-pigs by Weininger (1954), and also by Wilhelmj, Kierland and Owen (1962). These authors used an autologous freeze-pressed skin extract as antigen, which was thought to be a protein complex precursor of keratin.

Species variability to carcinogens has been studied and an attempt made to induce an autologous skin antibody in the guinea-pig, and to test the subsequent effect of skin painting with the carcinogen dimethylbenzanthrene (DMBA) (Anderson, 1967). In this experiment, it was thought that humoral antibody to the carcinogen hapten only occurred in the group of animals which had been sensitised to autologous skin before painting with the carcinogen. If this is so, the antibody which had been induced to skin components appears to have reacted with the skin-carcinogen complex during the months of painting and, subsequently, been able to combine with the hapten alone when tested by slide agglutination.

However, this does not appear to be the whole story, as it was possible in the guinea-pig to induce both carcinogen-protein binding and anti-target-organ antibody without inducing cancer. Indeed, it is thought that the response of either tolerance or hypersensitivity which was obtained was associated with the mechanism which prevents carcinogenesis in this species.

Both auto-immune diseases and, it is thought, malignant diseases are initiated by antibody attack in man. In the experiment quoted here, there is suggestive evidence that, in this system, it is possible to elicit an antibody to an organ (skin) and challenge that organ with a carcinogen, without inducing malignancy. The association in man between malignancy and hypersensitivity does not appear to be an alternative phenomenon as that of tolerance or hypersensitivity seems to be in the guinea-pig. The work of Grace and Dao (1959), Curtis, Blaylock and Harrell (1952) and of Curtis, Heckman and Wheeler (1961) on dermatomyosis and neoplasia demonstrated an immediate-type hypersensitivity to an autologous extract of tumour tissues, as well as a passive transfer reaction in a healthy volunteer. They suggested that tumour catabolic products were acting as a reagin which elicited a hypersensitivity response. However, it is unknown whether the association is causally related, either the cancer causing the associated disease or *vice versa*, or whether both diseases are an expression of some, as yet occult, process which generates both diseases. It is difficult to see how this point can be clarified experimentally, but consideration of another chronic disease, tuberculosis, suggests the possibility of tissue sensitisation.

It has been shown (Anderson, 1964) that a 20-methyl-cholanthrene-induced fibrosarcoma is capable of inducing an immune response in the spleen of the autologous tumour-bearing host. Passive cutaneous anaphylaxis constantly demonstrated in 31 guinea-pigs a specific immune response between autologous fibrosarcoma cells and autologous spleen cells. It was also found that when the spleen cells from an animal bearing a large tumour were used this specific recognition was not obtained. It was believed that the failure of specific recognition of tumour cells shown by spleen cells from animals bearing large tumours was due to a depletion of the immune mechanism. This observation is supported by the findings of Mikulski, Smith and Alexander (1964), that the growth rate of a chemically-induced tumour as transplant in syngeneic host rats is retarded if the tumour cells are mixed with spleen cells from a syngeneic donor which has been immunised against the tumour by injections of lethally irradiated tumour material. Also the protective effect was not obtained if the spleen was obtained from an animal bearing a large tumour. Maximum effect was obtained when the animal was killed 10–21 days after the tumour had been excised. An effort therefore was made to elucidate this fragmentary evidence for the presence of an antibody in neoplastic disease, and results have been obtained which point to an immediate type hypersensitivity response to preparations made from autologous tumours in some cases of human neoplastic disease. Anderson and Parsons (unpublished) found the injection of autologous tumour antigens into the skin of a patient with widespread cancer appears to cause a flare which is at maximum at about one hour and is faded by six hours. This flare is larger than that obtained to similar microsomes which had been heavily irradiated, or to autologous serum. Non-specific traumatic responses of the histamine-kinin type would appear to have been eliminated as a cause of the flare response by the very marginal response obtained to autologous serum. (However, care should be taken in accepting immediate type hypersensitivity responses in too unreserved a fashion, in view of the findings of Black (1963) who was able to suppress immediate-type hypersensitivity in allergic patients to the specific antigen by direct suggestion under hypnosis. Hence, there must also be some part of the immune mechanism under nervous control).

It is, indeed, theoretically logical that a graft-versus-host reaction initiates a defective immune response which itself prevents the effective operation of cellular responses. The position may be even more complicated than this suggests, in the light of the findings of Bielicky, Jezkova and Malina (1966) who examined the sera of patients with lupus erythematosis and found the highest titres of antibody in patients with the mildest forms of the disease. They concluded that auto-antibody is protective in this disease, and that there is an inverse correlation between manifestation of the disease and auto-antibody formation.

The fully neoplastic cell is believed to be eliciting antibody to normal, related cells, as suggested by Davis, Green and Timms (1961) who demonstrated a cold, incomplete, non-gamma globulin-type antibody on the red cell of patients with cancer, which was shown to have a larger than normal plaque size by electron microscopy studies and later to be of an a-2 type globulin. More recently, antibody consumption tests (Anthony and Parsons, 1965) have demonstrated the presence of a 1 gM or a 1 gG globulin on tumour cells, leucocytes of patients and animals with spontaneous, induced or transplanted tumours, which agrees with the work of Brody and Beizer (1963) who found antibody on both tumour cells and erythrocytes. Hence it appears that the *in vivo* neoplastic cell may emit either an antigen and an antibody or an antibody with antigenic specificity (which seems the more probable), against which the tumour-bearing host responds disastrously. The concept of a dedifferentiated cell producing antibody, or a malignant cell derived from tissues which are not normally believed to produce antibody doing so, is not surprising when the recent findings of Gurdon and Uehlinger (1966) are considered. He enucleated fertilised frog ova and substituted the nucleus from an intestinal cell and still obtained normally developed tadpoles. It is evident, therefore, that differentiated cells still retain potentialities which are not normally expressed. It is possible that the gammaglobulin which Anthony and Parsons (1965) found on tumour cells as well as on the leucocytes of patients may be occluding antigenic terminals on both normal as well as malignant cells, and may indeed be derived from both host and tumour cells. Hellman, Duke and Tucker (1965) studied the effect of thalidomide on homograft rejection in rats and in mice, and showed that if the host is treated with thalidomide there is a delay in the rejection of skin homografts. They also found that treating the skin graft with thalidomide, in a water bath for six hours before grafting, delayed graft rejection in these species. Whether there is any association between the delay in rejection of thalidomide-treated skin grafts and the delay in the induction of chemical carcinogenesis when the lymph nodes draining the area are irradiated remains to be seen (Anderson, 1963).

Dempster Calnan and Kulatilake (1963) showed that second set rejection of normal skin homografts could be prevented if the first sensitising graft were removed within eight days, and Vrubel and Vrubelova (1962) obtained homograft immunity in rabbits after modifying the lymph nodes draining the area of the graft. This suggests that a normal homograft emits an antigen-like substance which passes to the draining lymph nodes where it initiates an immune response. If malignant tissues bear any resemblance to normal tissues they may also emit such an antigen-like substance, which would be expected to act as a graft-versus-host reaction when the host immune response is grossly depleted or paralysed.

If tissue specific antigen can be deleted from the cell surface by factors extrinsic or intrinsic to the cell (by an antibody induced against the cell surface or faulty messenger RNA resultant on a genome alteration caused by irradiation or viral infection), this deletion should render the cell anonymous. However, the anonymity of the neoplastic cell is different from that of the chimeral cell, which is unrecognised by its host, and does not react against the host. It is believed that in the process of losing tissue specific antigens from the cell surface, the neoplastic cell becomes so altered that it reacts against the host from whom it induces a defective immune response, which is tumour promoting.

It is noteworthy, however, that not only does the skin of cancer patients persist longer than skin from healthy controls as a homograft on normal volunteers (Amos, Hattler and Shingleton, 1965), but normal skin is not rejected by patients in the terminal stages of cancer at the usual time (Morson, 1962). Hence it appears possible that not only is the immune system depleted in cancer patients, but the normal tissues of a cancer patient may be unable to express full antigenic potentiality.

It is postulated that there are two or more immune responses taking place in neoplasia, a host immune response against the tumour and a tumour-versus-host response. It is believed that it is the latter process, the tumour-versus-host response, which is responsible for the peculiar malignancy of neoplasia, indeed some chemotherapeutic agents may exert their beneficial effects by damping down or blocking the tumour-versus-host reaction. It has been demonstrated experimentally by Megirian (1965) that chlorambucil significantly delays the uptake of particulate matter from the blood stream by the reticulo-endothelial system. If chlorambucil blocks uptake of soluble antigens likewise, the possibility is further increased that this is the mechanism by which the drug exerts beneficial effect in neoplasia.

Consideration of the preceding observations regarding the interplay between host and tumour makes it appear likely that this relationship is integral to the understanding of the nature of neoplastic disease.

### SUMMARY

It is believed that two immune processes are taking place in established neoplastic disease, a host versus tumour response and a tumour versus host response, and that these two processes are integral to the disease.

### REFERENCES

Abercrombie, M. and Ambrose, E. J.—(1958) *Exp. Cell Res.*, **15**, 332.
Abercrombie, M. and Heaysman, J. E. M.—(1953) *Exp. Cell. Res.*, **5**, 111.
Amos, D. B., Hattler, B. G. and Shingleton, W. W.—(1965) *Lancet*, i, 414.
Anderson, M. R.—(1967) *J. Path. Bact.*, **92**, 591.—(1964) *Nature, Lond.*, **204**, 55.—(1963) *Nature, Lond.*, **198**, 599.
Anthony, H. M. and Parsons, M.—(1965) *Nature, Lond.*, **206**, 275.
Bielicky, T., Jezkova, Z. and Malina, L.—(1966) *Br. J. Derm.*, **78**, 29.
Black, S.—(1963) *Br. med. J.*, i, 990.
Brody, J. I. and Beizer, L. H.—(1963) *Blood*, **22**, 139.
Coman, D. R.—(1961) *Cancer Res.*, **21**, 1436.
Coman, D. R. and Anderson, T. F.—(1955) *Cancer Res.*, **15**, 541.
Curtis, A. C., Blaylock, H. C. and Harrell, E. R.—(1952) *J. Am. med. Ass.*, **150**, 844.
Curtis, A. C., Heckman, J. H. and Wheeler, A. H.—(1961) *J. Am. med. Ass.*, **178**, 571.
Davis, J. E., Green, H. N. and Timms, P. W.—(1961) *Nature, Lond.*, **191**, 923.
Dempster, W. J., Calnan, J. S. and Kulatilake, A. E.—(1963) *Br. med. J.*, i, 23.
Eisen, H. N., Orris, L. and Belman, S.—(1962) *J. exp. Med.*, **95**, 473.
Glaser, E. M., Spink, P. and O'Meara, R. A. Q.—(1965) *Nature, Lond.*, **208**, 1008.
Gordon, J.—(1964) *Nature, Lond.*, **203**, 884.
Grace, J. T. and Dao, T. I.—(1959) *Cancer, N.Y.*, **12**, 648.
Gurdon, J. B. and Uehlinger, V.—(1966) *Nature, Lond.*, **210**, 1240.
Hellman, K., Duke, D. I. and Tucker, D. F.—(1965) *Br. med. J.*, ii, 687.

HUMPHREY, J. H.—(1965) ' Molecular and Cellular Basis of Antibody Formation ', edited by J. Steizi. New York (Academic Press) p. 27.
MEGIRIAN, R.—(1965) *J. Reticuloendothelial Soc.*, **2**, 238.
MICHAELS, L.—(1964) *Lancet*, ii, 832.
MIKULSKI, Z. B., SMITH, C. AND ALEXANDER, P.—(1964) *Rep. Br. Emp. Cancer Campn.*, **42**, 46.
MORSON, B. C.—(1962) *J. Am.med. Ass.*, **179**, 316.
O'MEARA, R. A. Q. AND JACKSON, R. E.—(1958) *Ir. J. med. Sci.*, **391**, 327.
PARISH, W. E., CHAMPION, R. H. AND ROOK, A. J.—(1965) *Br. J. Derm.*, **77**, 479.
SULZBERGER, M. B., SHERWIN, R. W. AND HERMANN, F.—(1962) *J. invest. Derm.*, **39**, 179.
VRUBEL, J. AND VRUBELOVA, H.—(1962) *Nature, Lond.*, **193**, 186.
WEININGER, O.—(1954) *Science, N.Y.*, **119**, 285.
WEISS, L.—(1965) *Exp. Cell Res.*, **37**, 540.—(1960) *Int. Rev. Cytol.*, **9**, 220.
WILHELMJ, C. M., KIERLAND, R. R. AND OWEN, C. A.—(1962) *Archs. Derm.*, **85**, 161.
WOOD, S.—(1958) *Archs. Path.*, **66**, 550.
WOOD, S., HOLYOKE, E. D. AND YARDLEY, J. H.—(1961) Canad. Cancer Conf. IV, edited by Begg, R. W., Ham, A., Leblond, C. P., Noble, R. L. and Rossiter, R. J. New York, (Academic Press) p. 167.